# Self-assembly of PEGylated tetra-phenylalanine derivatives: structural insights from solution and solid state studies

**DOI:** 10.1038/srep26638

**Published:** 2016-05-25

**Authors:** Carlo Diaferia, Flavia Anna Mercurio, Cinzia Giannini, Teresa Sibillano, Giancarlo Morelli, Marilisa Leone, Antonella Accardo

**Affiliations:** 1 Department of Pharmacy and CIRPeB, University of Naples “Federico II”, via Mezzocannone 16, 80134 Napoli, Italy; 2 Institute of Biostructure and Bioimaging (IBB), CNR, via Mezzocannone 16, 80134 Napoli, Italy; 3 Institute of Crystallography (IC), CNR, Via Amendola 122, 70126 Bari, Italy

## Abstract

Water soluble fibers of PEGylated tetra-phenylalanine (F4), chemically modified at the N-terminus with the DOTA chelating agent, have been proposed as innovative contrast agent (CA) in Magnetic Resonance Imaging (MRI) upon complexation of the gadolinium ion. An in-depth structural characterization of PEGylated F4-fibers, in presence (DOTA-L_6_-F4) and in absence of DOTA (L_6_-F4), is reported in solution and at the solid state, by a multiplicity of techniques including CD, FTIR, NMR, DLS, WAXS and SAXS. This study aims to better understand how the aggregation process influences the performance of nanostructures as MRI CAs. Critical aggregation concentrations for L_6_-F4 (43 μM) and DOTA-L_6_-F4 (75 μM) indicate that self-aggregation process occurs in the same concentration range, independently of the presence of the CA. The driving force for the aggregation is the π-stacking between the side chains of the aromatic framework. CD, FTIR and WAXS measurements indicate an antiparallel β-sheet organization of the monomers in the resulting fibers. Moreover, WAXS and FTIR experiments point out that in solution the nanomaterials retain the same morphology and monomer organizations of the solid state, although the addition of the DOTA chelating agent affects the size and the degree of order of the fibers.

Peptide materials based on the aggregation of amphiphilic peptides represent a rapidly growing field within materials science^1^,^2^,^3^. They have been considered for several applications in different fields from electronic to nanomedicine. Amphiphilic peptides self-assemble in well-structured supramolecular materials as the result of an intricate network of interactions between hydrophobic and hydrophilic regions. The interaction mode can strongly influence both morphology and properties of the final peptide-based materials. Special interest was recently devoted to peptide self-assembling materials in which aggregation is promoted by aromatic amino acids, such as phenylalanine, tyrosine or tryptophan, where π-stacking interactions occur^4^,^5^,^6^. To this regard, a paradigmatic example is represented by the diphenylalanine (FF or F2) peptide, which constitutes the core recognition motif of Alzheimer’s β-amyloid peptide. FF is able to self-assemble into many different nanostructures from nanotubes to vesicles and organogels. Peptide nanostructures containing the FF motif or more extended aromatic sequences have been investigated for their mechanical, electrochemical and optical properties, and more recently for some nanomedicine applications^7^,^8^. Despite the growing literature about FF, only a few examples of new materials obtained by the self-assembling of tetraphenylalanines have been reported until now^9^,^10^,^11^,^12^. In order to get a deeper understanding of the molecular interactions involved in their self-assembly process, theoretical calculations for FFFF (also called F4) and Fmoc-FFFF aggregates have been recently reported^9^. Moreover, a few examples of tetraphenylalanine-polymer conjugates, in which the aromatic framework has been elongated at the N-terminus with addition of polymeric chains, have been proposed. The F4-PEG5000 derivative, synthetized by Hamley and coworkers, is able to self-aggregate in water solution at low concentrations (0.095 wt.%), whereas well-developed β-sheet structures occur only at higher concentration^10^. Others examples of F4-polymer conjugates (mPEO-F4-OEt), containing variable length of PEO chain (350, 1200 and 1800 Da) were synthesized by a click-chemistry reaction between an alkyne modified tetraphenylalanine (alkyne-F4-OEt) and the azide-terminated PEO oligomer (mPEO-N_3_). In a water/tetrahydrofuran mixture, mPEO-F4-OEt self-assembles initially in nanotubes (between 2 and 10 mg/mL) and successively, at higher concentration, the entanglement between adjacent nanotubes leads to the hydrogel formation^11^. The length of the hydrophilic block PEO influences significantly the assembling process by favouring fibers formation and worm-like micelles^12^. Very recently, we proposed the first example of PEGylated di-phenylalanine [DOTA(Gd)-L_6_-F2 and DTPA(Gd)-L_6_-F2] and tetra-phenylalanine [DOTA(Gd)-L_6_-F4 and DTPA(Gd)-L_6_-F4] conjugates as potential MRI CAs^13^. These conjugates contain two (F2) or four (F4) phenylalanine residues for self-assembly, a chelating agent, DOTA (1,4,7,10-tetraazacyclododecane-N,N,N,N-tetraacetic acid) or DTPA (diethylenetriamine pentaacetate), for achieving gadolinium coordination and an ethoxylic linker at six PEG units (L_6_) between the chelating group and the peptide region. All of these phenylalanine conjugates have been structurally characterized by CD, FTIR, fluorescence spectroscopies and X-ray diffraction on the dried fibers. Due to the steric hindrance of the bulk gadolinium complex, F2 conjugates were not able to keep their propensity to aggregate in water solution. On the contrary F4 adducts, with a more extended aromatic framework, kept their capability to aggregate after the synthetic modification with the Gd-complex. The replacement of the paramagnetic Gd(III) ion with others radioactive metal ions (^111^In, ^67/68^Ga) could open novel perspective for these nanostructures in others diagnostic fields. As an alternative, the chelating agent could be also replaced with an active pharmaceutical ingredient (API). However, at the best of our knowledge, until now it has not been fully explored how modifications on the flexible PEG chain could affect the molecular interactions governing the overall structural organization of the conjugates in aqueous solution. To this aim, here we report an in-depth comparative structural study of PEGylated tetra-phenylalanine nanostructures, in presence (DOTA-L_6_-F4) and in absence of DOTA (L_6_-F4), in solution and at the solid state. The work relies on a multidisciplinary approach including a variety of techniques such as Circular Dichroism (CD), Fourier Transform Infrared (FTIR), Dynamic Light Scattering (DLS), NMR, Wide-Angle (WAXS) and Small-Angle X-ray Scattering (SAXS). The combination of these techniques allows a full characterization of nanostructures both at the solid-state and in solution, clarifying their molecular organization also in solution at concentrations useful for MRI applications. These findings can help to improve the performance of poly-phenylalanine conjugates as contrast agents.

## Results and Discussion

### Synthesis and fluorescence spectroscopy

**Figure 1:**
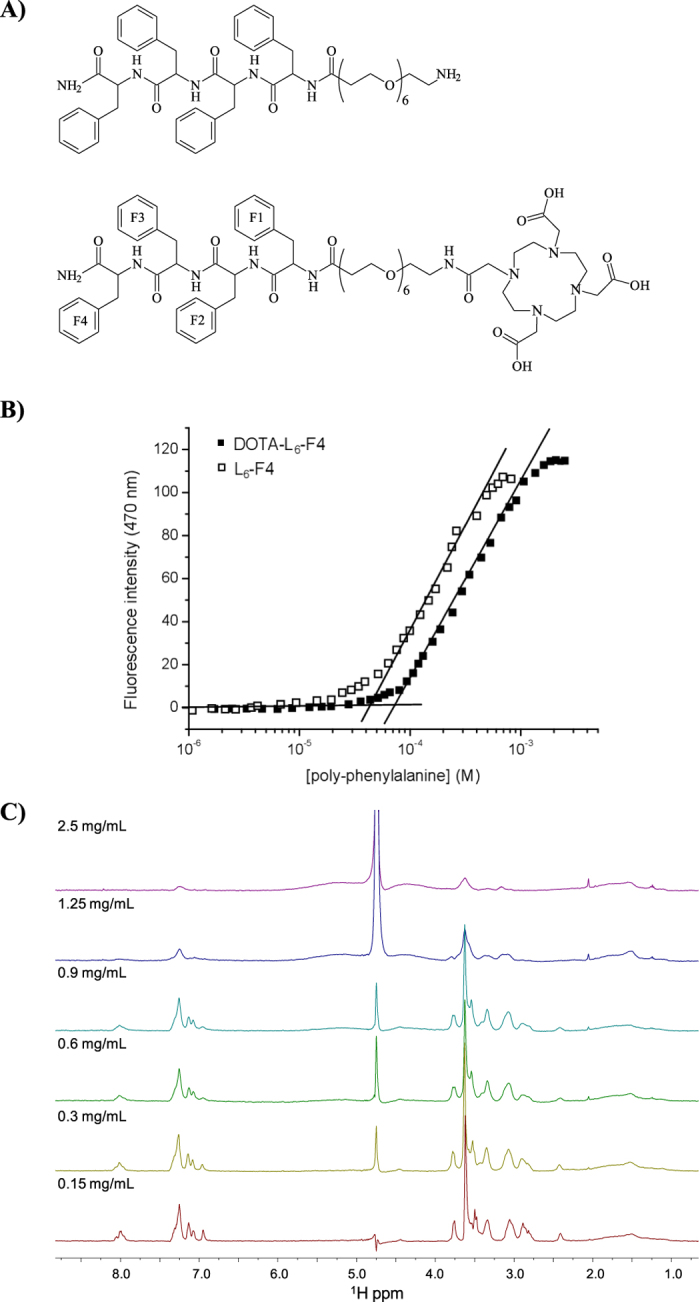
(**A**) Schematic representation of L_6_-F4 and DOTA-L_6_-F4 derivatives. (**B**) Fluorescence intensity emission of ANS fluorophore at 470 nm vs. concentration of L_6_-F4 and DOTA-L_6_-F4, CAC values are established from the break points. (**C**) 1D ^1^H NMR spectra of DOTA-L_6_-F4 recorded at 400 MHz and 298 K with samples dissolved in H_2_O/D_2_O 90/10 v/v at different concentrations.

PEGylated tetra-phenylalanine derivatives, L_6_-F4 and DOTA-L_6_-F4, are schematically reported in Fig. 1A. In DOTA-L_6_-F4, the DOTA bifunctional chelating agent, added at the N-terminus of the PEG moiety, can allow kinetically and thermodynamically stable coordination of radioactive or paramagnetic metal ions (^111^In, ^67/68^Ga, Gd(III)) for diagnostic applications in Nuclear Medicine or Magnetic Resonance Imaging^14^,^15^. Both peptides were obtained by Fmoc/tBu solid phase synthesis and subsequently purified by RP-HPLC chromatography. Critical aggregate concentration (CAC) values of L_6_-F4 and DOTA-L_6_-F4 were determined with a fluorescence-based method, in which 8-anilinonaphthalene-1-sulfonate ammonium salt (ANS) was used as fluorescent probe. Fluorescence emission of ANS is commonly observed at 460–480 nm only when this fluorophore is surrounded by a hydrophobic environment, such as in the micelle core^16^,^17^. The fluorescence intensity of an ANS solution (20 μM in aqueous solution) at 470 nm, as function of both tetra-phenylalanines concentration, is reported in Fig. 1B. The CAC values determined by Fig. 1B are 75 μM (~0.099 mg/mL) and 43 μM (~0.040 mg/mL) for DOTA-L_6_-F4 and L_6_-F4, respectively. For L_6_-F4 the CAC value is in good agreement with that of ~167 μM, 0.095 wt.%, previously measured by Hamley *et al*., through pyrene fluorescence assays, for another PEGylated tetra-phenylalanine (F4-PEG5000)^10^. As expected, the CAC of L_6_-F4 is slightly lower than the CAC of F4-PEG5000, in which the hydrophobic/hydrophilic ratio is more unbalanced towards hydrophilic share. Moreover, the CAC values for L_6_-F4 and DOTA-L_6_-F4 are quite similar, thus indicating that the self-aggregation process occurs in the same range of concentration, independently of the presence of the chelating agent at the N-terminus. Undoubtedly, the driving force for the aggregation is the π-stacking between the side chains of the aromatic framework. In this perspective, it does not surprise that the two peptides, containing the same aromatic portion, show a very close CAC value. However, it is also well-known that chelating agents such as DOTA and DTPA, can cause a steric and/or electrostatic hindrance, thus providing an increase of the CAC value^13^,^18^. In our case, we can ascribe the low incidence of DOTA macrocycle on the fibrillary process in presence of the PEG chain; the latter being long enough to act as a spacer between the chelating agent and the aromatic framework.

### NMR spectroscopy

**Figure 2:**
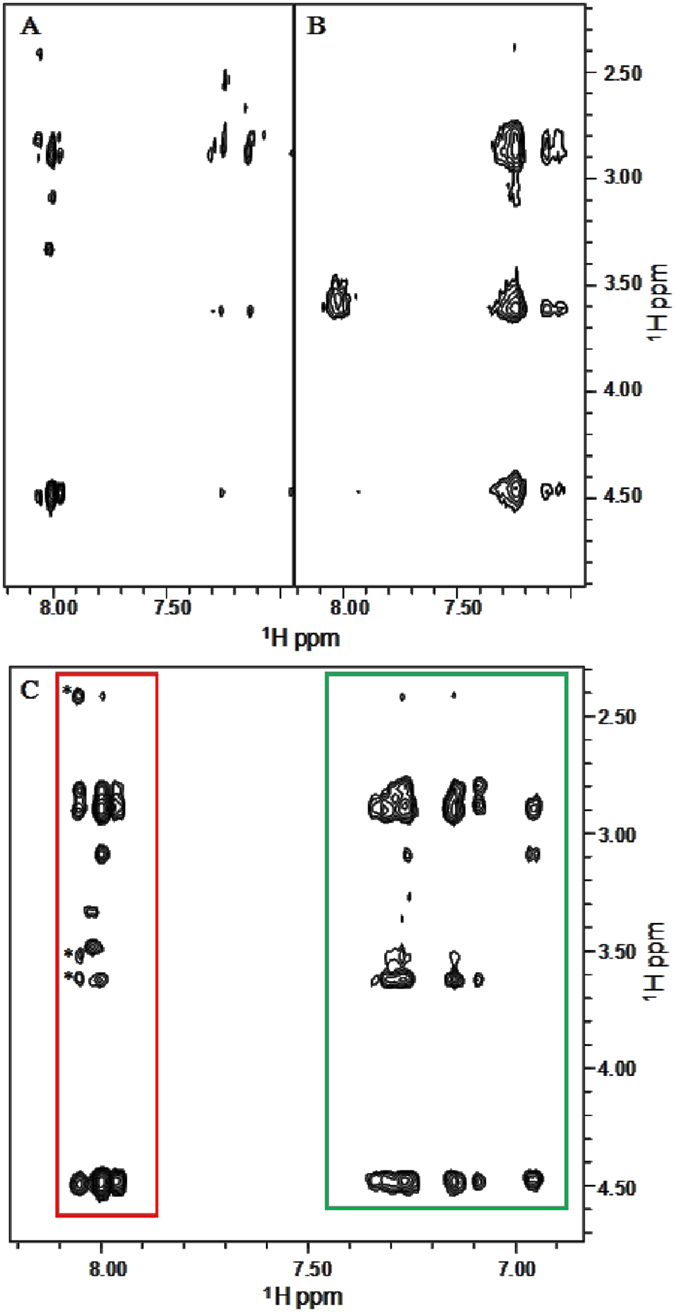
Comparison of 2D [^1^H, ^1^H] NOESY 300 spectra of DOTA-L_6_-F4 at concentrations equal to 0.11 mM (0.15 mg/mL) (**A**) and 0.94 mM (1.25 mg/mL) (**B**). 2D NOESY 300 spectrum of DOTA-L_6_-F4 (0.34 mM, 0.45 mg/mL) (**C**). NOE contacts involving H_N_ protons with side chain protons are included in the red rectangle whereas correlations arising from aromatic protons are incorporated in the green box. The *indicates a correlation between the H_N_ amide proton of F1 and close linker (L_6_) protons (**C**).

NMR spectroscopy was implemented to better investigate the conformational properties of DOTA-L_6_-F4 in solution. Due to the high tendency of the compound to aggregate, experiments at different concentrations were conducted to get an estimate of the critical aggregation concentration (CAC). 1D [^1^H] experiments were first acquired (Fig. 1C). At a concentration equal to 0.9 mg/mL (680 μM) or higher, aggregation phenomena resulted so relevant that extensive line broadening dominated the NMR spectra causing loss of signal intensity (Fig. 1C). The sensitivity of the NMR experiments highly improved by lowering the concentration from 0.9 mg/mL (680 μM) to 0.3 mg/mL (230 μM). A further dilution to 0.15 mg/mL (110 μM) caused only a slight enhancement of the spectrum. These results allowed us to set the CAC below 0.15 mg/mL. In addition, this aggregation propensity was confirmed by means of 2D [^1^H, ^1^H] spectroscopy. 2D [^1^H, ^1^H] NOESY experiments^19^ were collected at three different concentrations (Fig. 2): spectra of diluted F4-L_6_-DOTA samples (concentrations equal or below 110 μM) (Fig. 2A) contained only a few cross-peaks and reflected the almost complete absence of aggregation phenomena and the presence in solution of small, fast tumbling flexible species (Fig. 2A). On the contrary, at a concentration equal to 1.25 mg/mL (0.94 mM), NOESY spectra were characterized by extensive signal enlargement, due to the occurrence of large size aggregates in solution (Fig. 2B). The consequent low signal intensity, caused by high aggregation levels, did not allow conducting a detailed structural characterization of the concentrated DOTA-L_6_-F4 samples containing large size aggregates.

**Figure 3:**
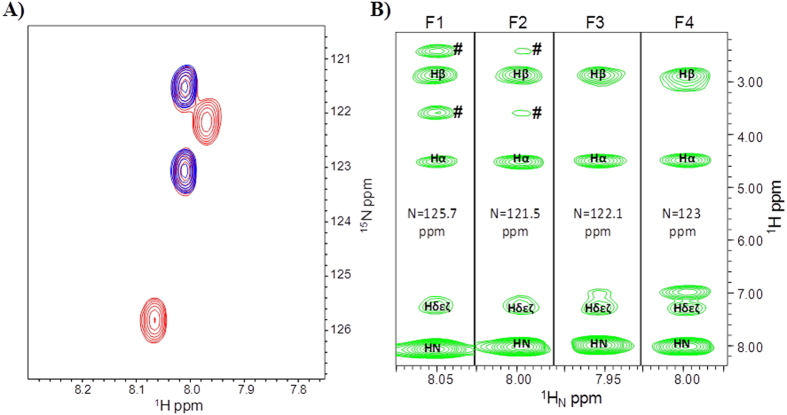
Overlay of [^1^H,^15^N] HSQC spectra of DOTA-L_6_-F4 (100 μM concentration) with ^15^N/^13^C double-labeled F(1,2,3,4) (red) or F(2,4) (blue) (**A**). Strips from ^1^H/^1^H_N_ slices of the 3D ^15^N resolved-[^1^H, ^1^H] NOESY-HSQC spectrum of ^15^N,^13^C F(1,2,3,4) labeled DOTA-L_6_-F4 (500 μM concentration). Each strip corresponds to a single Phe residue as indicated at the top of each slice. Aromatic protons are reported as Hδεζ. The ^#^highlights unambiguous NOEs between the backbone H_N_ of F1 and F2 and the closest -NHCOC**H**_2_C**H**_2_O- protons of the L6 linker portion (**B**). Spectra were recorded at 600 MHz and 298 K.

**Figure 4:**
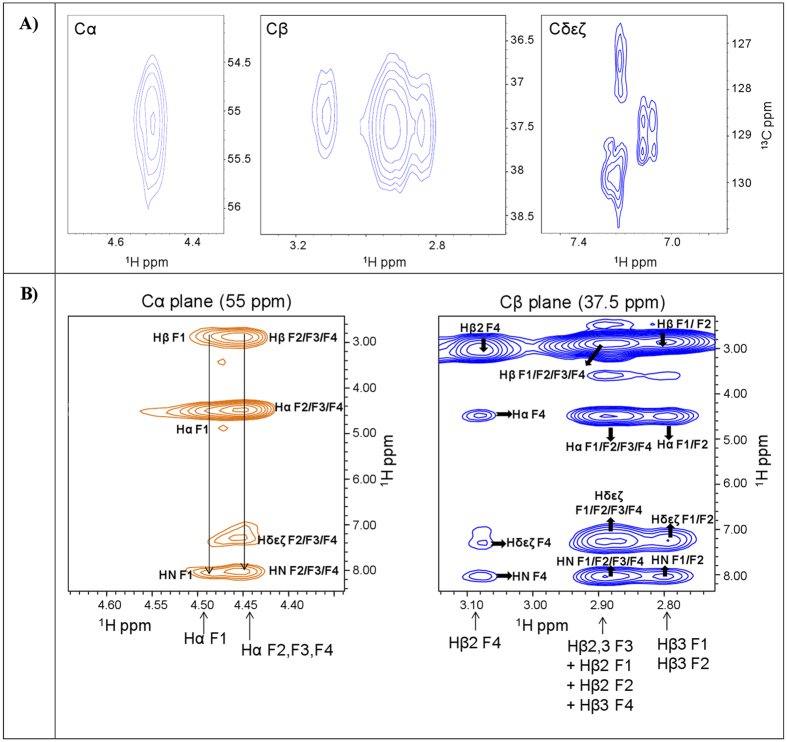
[^1^H, ^13^C] HSQC spectra of DOTA-L_6_-F4 (100 μM), recorded with a sample containing ^15^N/^13^C double-labeled F(1,2,3,4) (**A**). The Cα, Cβ and aromatic region of the [^1^H, ^13^C] HSQC spectrum are reported in the left, middle and right panels respectively (**B**). A few [^1^H, ^1^Hα] (left side) and [^1^H, ^1^Hβ] (right side) strips from the 3D ^13^C resolved-[^1^H, ^1^H] NOESY-HSQC spectrum of double ^15^N/^13^C F(1,2,3,4) labeled DOTA-L6-F4 are shown. Assignments are reported for each Phe residue.

To get insights into the secondary structure elements characterizing small size aggregates formed by DOTA-L_6_-F4, NMR spectra were recorded at concentrations between 300 and 500 μM, because under these experimental conditions good quality solution NMR spectra could be collected (Fig. 2C). Proton resonance assignments for DOTA-L_6_-F4 were obtained by means of a canonical protocol based on comparison of 2D TOCSY and NOESY experiments^20^ (Supporting Table S1). Sequential assignments for the four phenylalanine residues were further confirmed by means of heteronuclear 2D and 3D experiments (Figs 3 and 4) recorded with double labeled ^15^N/^13^C DOTA-L_6_-F4 containing either selective F(2,4) or uniformly F(1,2,3,4) labeling.

The H_N_ chemical shifts for the four phenylalanine residues are distributed in a very narrow chemical shift range centered around 8 ppm, while, Hα protons resonate at almost identical chemical shifts (Fig. 3 and Table S1). Differences between observed Hα chemical shifts and random coil values were small (Supporting Figure S1)^21^, thus indicating that at the concentration used to run the experiments (i.e., 0.3–0.5 mM range) random coil species were prevalent. Extensive chemical shifts degeneracy could be revealed also for ^13^Cα, ^13^Cβ and aromatic carbon atoms of the four phenylalanine residues (Fig. 4). Previous studies have demonstrated that, in the solid state, DOTA-L_6_-F4 forms ordered aggregates, characterized by an antiparallel β-sheet structure^13^. We carefully analyzed 2D [^1^H, ^1^H] NOESY (Fig. 2C), 3D ^15^N resolved-[^1^H, ^1^H] NOESY-HSQC (250 ms mixing time) (Fig. 3B), and 3D ^13^C resolved-[^1^H, ^1^H] NOESY-HSQC (200 ms mixing time) (Fig. 4B), trying to confirm the presence of such ordered species in the solution state. However, as already specified, at the concentrations exploited by NMR, the aggregation level was possibly low and the equilibrium between ordered and disordered species in solution was moved towards the latter forms as clearly indicated by Hα CSD values (Figure S1)^21^.

It’s also worth noting that Zanuy and coworkers^22^ have previously reported a computational conformational study on polyphenylalanine containing peptides and on their PEG adducts. Theoretical quantum mechanical calculations indicated a low tendency of the isolated tetra-phenylalanine (F4) peptide to adopt a fully extended β-conformation whereas pointed out the highest stability of ribbon-like organizations, made up of regular propagation of γ-turns motives through residues 1 to 4, or helical-like structures composed of alternated repetitions of canonical and reverse γ-turns^22^. Once the PEG unit was added to the F4 peptide, molecular dynamics simulations performed in water starting from a fully extended F4 conformation, indicated that the peptide unit of the resulting material tended to maintain the conformational preferences of the isolated F4 portion and thus to assume a more folded structural organization similar to the pseudo-ribbon or helical arrangements described above. However, this previous computational analysis^22^ was carried out on monomeric peptide units without taking into account aggregation effects in solution, that even at low extent, may indeed stabilize the fully extended structures.

For our DOTA-L6-F4 compound, chemical shifts degeneracy among atoms in the four phenylalanine residues made also impossible to unambiguously distinguish intra- from inter-molecular contacts as well as intra- from inter-residue NOEs (Figs 3B and 4B) that could have clearly witnessed the presence of an organized β-structure. Nevertheless, we can only realistically speculate that the DOTA-L_6_-F4 molecules preferentially adopt an extended structure. Indeed, NOE effects between linker L_6_ protons and phenylalanine atoms are limited to short range contact involving primarily F1 and the closest -NHCOC**H**_2_C**H**_2_O- protons of the linker L_6_ (Figs 1A and 2C). 2D [^1^H, ^1^H] spectra were recorded also to compare the conformational behavior of DOTA-L_6_-F4 and L_6_-F4 (Supporting Figures S2). In detail, no chemical shifts changes in the 4F- portion of the two molecules could be revealed by analysis of TOCSY spectra (Figure S2). This indicates that the addition of a chelating agent -like DOTA- does not influence the conformational behavior.

### DLS characterization

Hydrodynamic radii (R_H_) and diffusion coefficients (*D*) of L_6_-F4 and DOTA-L_6_-F4 in water solution were measured by DLS at 2.0 mg/mL. Both samples show a mono-modal distribution due to translational diffusion process of nanostructures (data not shown). The time correlation functions of the scattered intensity g(2)(t)-1 for both tetra-phenylalanine derivatives are reported in Supporting Figure S3A. DLS measurements for L6-F4 and DOTA-L_6_-F4 nanostructures at different concentrations (2.0, 5.0 and 10 mg/mL) were also performed in order to investigate the effect of the concentration on the size of nanostructures and the intensity correlation functions for L6-F4 are reported in Figure S3B.

Data reveal a translation of the correlation function for DOTA-L_6_-F4 at longer decay time with respect to L_6_-F4, with apparent translational diffusion coefficients *D* = (1.8 ± 0.1) × 10^−12^ and (7.2 ± 0.3) × 10^−12^ m^2^ s^−1^ respectively. These data were directly correlated to the apparent hydrodynamic radii (77 and 300 nm for L_6_-F4 and for DOTA-L_6_-F4) through the Stokes-Einstein equation. These R_H_ values are compatible with supramolecular aggregates with elongated shape^23^ such as open bilayers, worm-like micelles or nanofibers as in this case. The slower motion of DOTA-L_6_-F4 with respect to L_6_-F4 suggests that, at a similar concentration, nanostructures containing the DOTA chelating agent have a higher propensity to self-aggregate, with respect to the corresponding derivative in absence of DOTA. This hypothesis is also supported by the DLS analysis of nanostructures at different concentration (Figure S3B).

### Secondary structure

**Figure 5:**
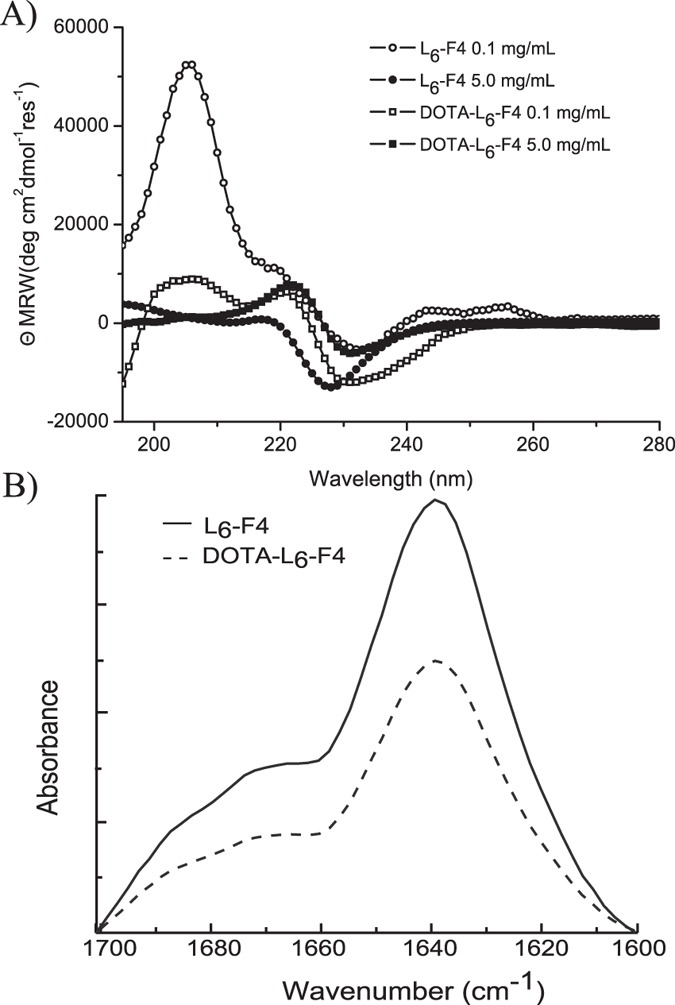
Spectroscopic characterization of L_6_-F4 and DOTA-L_6_-F4 peptide derivatives by CD and FTIR. (**A**) Far-UV CD spectra of both peptides in aqueous solution at (5.0 and 0.1 mg/mL) recorded between 280 and 195 nm; (**B**) FTIR spectra in the amide I region at 2.0 mg/mL concentration.

Structural characterization of these nanostructures by NMR spectroscopy fails in a range of concentration above 500 μM. In order to deeply investigate the aggregation properties in this range of concentrations, we studied the secondary structure of these peptide derivatives by CD and FTIR spectroscopies. CD spectra of L_6_-F4 in solution, recorded between 280 and 195 nm, are reported in Fig. 5A. For comparison, the dichroic tendency of DOTA-L_6_-F4, previously studied by us^13^ in the same experimental conditions and concentrations has been also reported. CD spectra of PEGylated tetra-phenylalanine L_6_-F4, at concentrations close to the CAC value (0.1 mg/mL), show two maxima (at 205 and 220 nm) and a minimum at 232 nm. The two maxima can be attributed to aromatic side-chains stacking, whereas the minimum can be associated with a β-structure^13^. Significant variations of the dichroic signal can be observed at 5 mg/mL, which corresponds to a concentration higher than the CAC value: the main minimum at 230 nm and the complete absence of the maximum at 205 nm can be ascribed to a dominant β-sheet arrangement^10^,^11^,^12^. For each concentration, CD spectra of tetra- phenylalanine in presence of the DOTA macrocycle did not show significant differences in the dichroic tendency. However, according to literature^11^, the spectrum of L_6_-F4 at 0.1 mg/mL shows a higher intensity of the maximum at 205 nm with respect to DOTA-L_6_-F4. However, the spectra of the two aromatic molecules here described show high similitude, thus suggesting that the addition of the chelating agent at the N-terminus of the sequence does not affect their assembling properties in solution. Further information on the secondary structure adopted by L_6_-F4 and DOTA-L_6_-F4 in solution (2.0 mg/mL) was obtained using FTIR spectroscopy in the amide I region (see Fig. 5B). Both spectra show two peaks at 1637 cm^−1^ and 1680 cm^−1^, respectively. The peak at 1637 cm^−1^ is strongly indicative of β-sheet formation, whereas the lower intensity of the second peak at 1680 cm^−1^ is indicative of an antiparallel orientation of the β-sheets^24^. FTIR spectra recorded on dried film of both peptides showed a similar profile with respect to samples in solution (data not shown).

### Wide-Angle and Small-Angle X-ray Scattering

**Figure 6:**
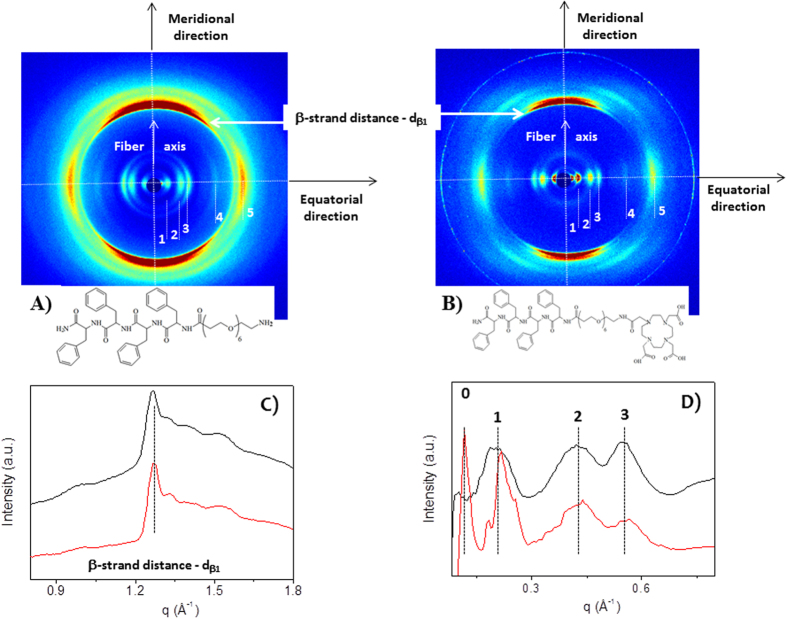
(**A**,**B**) show the two-dimensional (2D) WAXS pattern collected on the L_6_-F4 and DOTA-L_6_-F4 dried samples, respectively; (**C**,**D**) show the 1D profiles corresponding to the L_6_-F4 (black) and DOTA-L_6_-F4 (red).

Figure 6(A,B) present the two-dimensional (2D) WAXS pattern collected on the L_6_-F4 and DOTA-L_6_-F4 dried samples, respectively. The 2D patterns, once centered, calibrated and radially folded into 1D profiles by integrating along the azimuth, are displayed in Fig. 6(C,D) in the 0.8–1.8 Å^−1^ and 0.08–0.8 Å^−1^ q-ranges, respectively (black corresponding to the L_6_-F4 and red to DOTA-L_6_-F4). The 2D patterns both display the typical cross-β fiber diffraction pattern which contains, along the meridional and equatorial directions, the following fingerprints:

i) the meridional reflection at d_β1_ = 4.9 ± 0.3 Å, marked by the white arrow in Fig. 6(A,B), which corresponds to the highest peak in Fig. 6(C). The meridional reflections are due to the separation between adjacent peptide backbones organized into β-strands along the fiber axis. In the meridional direction we have not measured any diffraction reflection at 2d_β1_ = 9.8 Å. This absence could be either due to the predominance of parallel β-strands organization or to the 2_1_ symmetry of antiparallel β sheets^25^. However, the presence of the peak at 1680 cm^−1^ in the FTIR spectra, recorded on both tetra-phenylalanines in solution (Fig. 5) and dried sample, led us to hypothesize an antiparallel β sheet with 2_1_ symmetry.

ii) A series of equatorial reflections were marked by the dotted lines labelled as 1, 2 3, 4, 5. The most intense equatorial lines (1, 2, 3) correspond to the spacing: d_1_ = 31 ± 1 Å, d_2_ = 15 ± 1 Å = d_1_/2, d_3_ = 11 ± 0.3 Å, indicating the presence of a lamellar phase along the fiber cross-section. Only in the case of the DOTA-L_6_-F4, another equatorial reflection is measured at very small q value, labelled as 0 in the red pattern of Fig. 6D. This reflection corresponds to a spacing d_0_ = 54 ± 4 Å, although this evaluation is less accurate being the reflection on the tail of the primary beam. The d_0_ and d_1_ could match with the lengths of the backbones for the DOTA-L_6_-F4 and L_6_-F4, respectively (http://pubchem.ncbi.nlm.nih.gov), taking into account a possible folding of the PEG chain.

iii) For both samples the 2D WAXS data are due to oriented fibers, as evidenced by the presence of arcs instead of fully rings. Diffraction patterns, made up of longer arcs, indicate higher fiber disorder (mosaicity effect). Indeed, Figure S4 of the Supporting Information displays four fiber diffraction 2D patterns, simulated by the *CLEARER* program^26^, for increasing fiber disorder: 0.1 rad (5.7°) in (A), 0.2 rad (11.4°) in (B), 0.5 rad (28.6°) in (C) and 0.75 rad (43°) in (D). The longer arcs of the L_6_-F4 sample with respect to the DOTA-L_6_-F4 prove the presence of a larger disorder for the L_6_-F4 sample. Comparing with the simulated patterns, data registered for the DOTA-L_6_-F4 can be well described by a fiber disorder as small as 0.1 rad, while for the L_6_-F4 sample the longer arcs can be explained by a disorder of about 0.2 rad. This is confirmed also by the full-width-at-half-maximum of the meridional and equatorial peaks which decreases for the DOTA-L_6_-F4, proving how the additional DOTA produces fibers with a higher order degree. It is worth noting that a very large disorder in the fiber would cause the diffraction pattern to lose the typical characteristic arcs which degenerate in the full rings of a powder-like sample (Figure S4D). This behavior was observed by Hamley *et al*.^10^ for F4-PEG5000, in which the long PEG moiety causes crystallization effects.

2D SAXS data were also collected on the same L_6_-F4 and DOTA-L_6_-F4 dried fibers and shown in Figure S5A,B of the Supporting Information, respectively. The data were centered, calibrated and folded in the 1D patterns of Fig. 5C,D, respectively. The SAXS data confirm and reinforce the previous findings. Indeed, while the L_6_-F4 sample does not present any SAXS diffraction, the pattern registered on DOTA-L_6_-F4 clearly shows fiber diffraction partial rings. This is a distinct indication that the DOTA chelating agent increases the fiber order, extending it from atomic to nanoscale.

**Figure 7:**
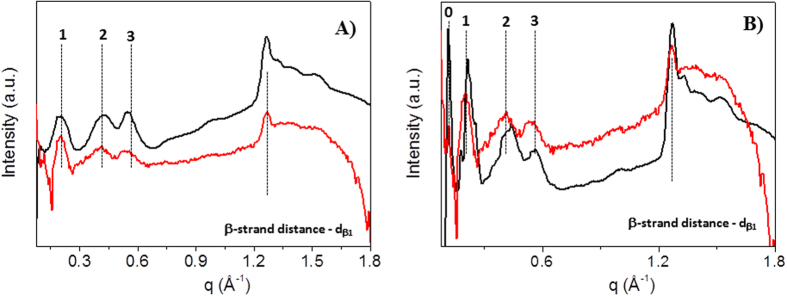
(**A**,**B**) show the WAXS profiles, displayed in red, collected on the L_6_-F4 and DOTA-L_6_-F4 solutions (25 mg/mL) to be compared with the profiles obtained on the analogous dried samples (black line).

Finally, Fig. 7(A,B) show the WAXS profiles collected on the L_6_-F4 and DOTA-L_6_-F4 solutions (25 mg/mL), respectively, after subtraction of the water contribution (red profiles). In each of them the pattern was compared with the analogous measured in the dried samples (black profiles). The comparison allows identifying exactly the same fingerprints measured for the dried fibers, bringing to the same conclusions. Also in the case of solutions the DOTA-L_6_-F4 sample shows sharpest peaks, with respect to the L_6_-F4 one, and therefore a more ordered lattice.

### Structure and relaxometric properties

DOTA-L_6_-F4, and its analogue DTPA-L_6_-F4, have been previously complexed with the paramagnetic gadolinium ion and studied for their relaxometric behavior with the aim to be used as positive MRI contrast agents (CAs)^13^. In principle, the efficacy of a MRI CAs depends on the ability to enhance the water protons relaxation rate in aqueous solutions due to the magnetic dipolar interaction between unpaired electrons on the gadolinium ions and the water protons, which is usually defined as longitudinal “relaxivity” (r_1p_) and is referred to the water proton relaxation rate of a solution containing the Gd-complex at one millimolar concentration. The relaxivity of a Gd-containing system depends on the complex interplay of structural, dynamic and electronic parameters. At the frequencies most commonly used in commercial tomographs (20–60 MHz), r_1p_ is generally determined by the reorientational correlation time (τ_R_) of the chelate so that high molecular weight systems display higher relaxivity. Moreover, when the gadolinium containing adduct is organized in a supramolecular structure with a well-defined three-dimensional order, slow reorientational correlation times and, consequently, high relaxivity values are expected.

This is the case for DOTA(Gd)-L_6_-F4, and its analogue DTPA(Gd)-L_6_-F4 that present relaxivity values, measured at 21.5 MHz (0.5 T) and 298 K, of 14.8 and 14.0 mM^−1^s^−1^, respectively^13^. These values, that are about three times higher the ones measured for the free monomeric DOTA(Gd) and DTPA(Gd) complexes in water solutions under the same experimental conditions, could be related to the structural organization observed for DOTA-L_6_-F4. Under the experimental conditions of the MRI studies, DOTA-L_6_-F4 monomers aggregate in well-ordered and very stable fibers and the driving force of aggregation is represented by the π-stacking contacts between the side chains of the aromatic framework. CD, FTIR, NMR and WAXS measurements, here reported, confirm the capability of the monomers to self-aggregate in fibers with an antiparallel β-sheet organization both in solution and at the solid state. In particular, NMR and WAXS results highlight the presence of highly stable nanofibers yet in solution, thus suggesting a potential application of these compounds as MRI contrast agents. Moreover, the herein reported in deep physicochemical characterization of these materials allows to unequivocally define the structural parameters characterizing the fibers, such as the inter-planar distances and the L6 folding around the fibers. The knowledge of these parameters can help clarifying important aspects to improve the performances of the final MRI contrast agent, in term of relaxivity values. For example, the mobility of the DOTA chelating agent along the linker spacer, with respect to the overall fibril-like structure, influences the τ_R_ values that result quite short if compared to those usually found for nano-sized aggregates^27^,^28^. Our data clearly indicate that to enhance the performances of DOTA(Gd)-L_6_-F4 fibers as MRI contrast agents, an increase of the rigidity of the DOTA moiety, pending from the fiber organization of DOTA-L_6_-F4 should be achieved.

## Conclusions

Extraordinary interest was recently devoted to peptide materials containing aromatic amino acids in which the aggregation process is promoted by π-stacking interactions. Very recently, we proposed novel PEGylated-F4 fibers, highly soluble in water solution, as potential CAs in MRI. The PEGylated-F4 was chemically modified at the N-terminus with branched DTPA or macrocyclic DOTA chelating agents, both of them able to allow kinetically and thermodynamically stable coordination of metal ions for diagnostic purpose. The comparative structural study of PEGylated tetra-phenylalanine fibers, in presence (DOTA-L_6_-F4) and in absence of DOTA (L_6_-F4), points out the high similitude between the resulting nanomaterials. Both nanofibers show an antiparallel β-sheets organization, although in DOTA-L_6_-F4 fibers an increase in the size and in the order degree was observed. This result suggests the low incidence of the DOTA macrocycle on the β-sheets organization, as a consequence of the PEG chain, the latter being long enough to place a distance between the chelating agent and the aromatic framework. Nevertheless it remains to understand, at the molecular level, what is the rationale for the higher inner order of DOTA-L_6_-F4 nanostructures with respect to L_6_-F4 ones. The organization in well-ordered and stable fibers of the DOTA-L_6_-F4 monomers, observed by comparing structural data from a multiplicity of experimental techniques (CD, FTIR, NMR, DLS, WAXS and SAXS) is responsible of the three-fold increase in the relaxivity value of DOTA(Gd) complex. As a consequence, a potential use of these nanofibers as MRI contrast agent can be realistically envisioned. On the other hand, the structural characterization here reported confirms and reinforces our previous hypothesis about the high flexibility of the DOTA moiety, bound to a quite long PEG chain. A more rigid organization should be advantageous to increase the relaxometric performance of the contrast agent. Despite this issue remains to be improved, the structural similitude of these nanofibers with the fibrillary oligomers (AβOs) or plaques commonly observed during the progression of several neurodegenerative disorders (Alzheimer’s, Parkinson’s and prion-related diseases) open new perspectives towards the development of diagnostic tools for their early detection. Moreover, we can realistically suppose that the replacement of DOTA with another active pharmaceutical ingredient, characterized by a comparable steric and electrostatic hindrance, would not cause significant structural differences. Based on these findings we can conclude that L_6_-F4, for its handily preparation and high water solubility, can represent a promising building block for the synthesis of novel materials for therapeutic or diagnostic applications.

## Materials and Methods

### Peptide synthesis

L_6_-F4 and DOTA-L_6_-F4 peptides were synthesized by solid phase synthesis and purified by RP-HPLC chromatography, according to the procedure previously described^13^. Similarly, double labeled ^15^N/^13^C DOTA-L_6_-F4 containing either selective (Phe 2,4) or uniformly Phe(1,2,3,4) labeling were synthesized by replacing Fmoc-Phe-OH with Fmoc-Phe-OH-^13^C_9_,^15^N. Double labeled phenylalanine was purchased by Sigma Aldrich (Milan, Italy).

### Fluorescence studies

CAC values were obtained by fluorescence measurements. Fluorescence spectra were recorded at room temperature on a Jasco Model FP-750 spectrofluorophotometer in a 1.0 cm path length quartz cell. Equal excitation and emission bandwidths (5 nm) were used throughout the experiments with a recording speed of 125 nm/min and automatic selection of the time constant. The CAC values were measured by using 8-anilino-1-naphthalene sulfonic acid ammonium salt (ANS) as fluorescent probe^29^,^30^. Small aliquots of peptide derivatives in water solutions were added to 1.0 mL of aqueous solution of ANS (20 μM). Final spectra, to be used for calculations, were obtained after blank correction and adjustment for dilution. The ANS fluorescence intensity was followed as a function of the peptide concentration. The CAC values were determined, upon excitation at 350 nm, by linear least-squares fitting of the fluorescence emission at 470 nm versus the poly-phenylalanine concentration, as the crossing point between the changes of slope.

### NMR experiments

NMR experiments were acquired at 298 K on either a Varian Unity Inova 600 MHz spectrometer equipped with a cold probe or a 400 MHz Varian instrument provided with a 5-mm triple resonance probe and z-axis pulsed-field gradients. All samples were dissolved in a mixture H_2_O/D_2_O (98% D, Armar Chemicals, Dottingen, Switzerland) 90/10 v/v with a total volume equal to 600 μL. The L_6_-F4 sample (0.05–1.5 mM concentration range) was analyzed through a set of 2D experiments: 2D [^1^H, ^1^H] TOCSY^31^ (70 ms mixing time), 2D [^1^H, ^1^H] NOESY^19^ (300 ms mixing time), and 2D [^1^H, ^1^H] ROESY (Rotating frame Overhauser Enhancement Spectroscopy)^32^ (150 and 250 ms mixing times). For the DOTA conjugated molecule DOTA-L_6_-F4 1D [^1^H] experiments were registered at the following concentrations: 1.9, 0.9, 0.7, 0.4, 0.2, 0.1 mM; whereas 2D [^1^H, ^1^H] TOCSY (70 ms mixing time), and 2D [^1^H, ^1^H] NOESY (300 ms mixing time) were acquired at 0.1, 0.3, and 0.9 mM.

1D spectra were usually acquired with a relaxation delay d1 of 1.5 s and 32–512 scans; 2D experiments were recorded with 16–64 scans, 128–256 FIDs in t1, 1024 or 2048 data points in t2. Further 3D and 2D heteronuclear correlation experiments^33^,^34^ (i.e., 2D [^1^H, ^15^N] HSQC (Heteronuclear Single Quantum Correlation Spectroscopy), and 2D [^1^H, ^13^C] HSQC, 3D ^15^N resolved-[^1^H, ^1^H] NOESY-HSQC (250 ms mixing time), 3D ^13^C resolved-[^1^H, ^1^H] NOESY-HSQC (200 ms mixing time), were set up with two different ^15^N/^13^C Phe labeled DOTA-L_6_-F4 samples (concentration range 0.1–1 mM): sample A) containing all phenylalanine residues uniformly labeled with ^13^C and ^15^N and sample B) containing instead only ^15^N/^13^C double labeled F2 and F4 (Fig. 1A). The process of proton resonance assignment was performed by following a canonical protocol based on comparison of TOCSY, NOESY and ROESY experiments (See Table S1)^20^.

Water suppression was achieved by Excitation Sculpting^35^. Spectra were processed with VNMRJ (Varian by Agilent Technologies, Italy) and analyzed with NEASY^36^ comprised in the CARA software package (http://www.nmr.ch/). 2D HSQC spectra were compared with the program Sparky (T. D. Goddard and D. G. Kneller, SPARKY 3, University of California, San Francisco). Chemical shift deviations from random coil values for Hα protons (CSD) were calculated with the protocol suggested by Kjaergaard and collaborators by keeping into account the influence of neighboring amino acids^21^,^37^,^38^.

### DLS measurements

Hydrodynamic radii (R_H_) and diffusion coefficients (*D*) of tetra-phenylalanine nanostructures were measured by Dynamic Light Scattering technique (DLS). DLS measurements were carried out using a Zetasizer Nano ZS (Malvern Instruments, Westborough, MA) that employs a 173° backscatter detector. Other instrumental settings are measurement position (mm): 4.65; attenuator: 8; temperature: 25 °C; cell: disposable sizing cuvette. DLS measurements in triplicate were carried out on aqueous samples at 2.0 mg/mL, after centrifugation at room temperature at 13,000 rpm for 5 minutes.

### Circular Dichroism

Far-UV CD spectra of the peptide derivatives in aqueous solution were collected on a Jasco J-810 spectropolarimeter equipped with a NesLab RTE111 thermal controller unit using a 0.1 mm quartz cell at 25 °C. The spectra of samples at (0.1 and 5.0 mg/mL) are recorded from 280 to 195 nm. Other experimental settings were: scan speed = 10 nm/min; sensitivity = 50 mdeg; time constant = 16 s; bandwidth = 1 nm. Each spectrum was obtained by averaging three scans and corrected for the blank. Here Θ represents the mean residue ellipticity (MRE), i.e. the ellipticity per mole of peptide divided by the number of amino acid residues in the peptide.

### Fourier Transform Infrared spectroscopy (FTIR)

FTIR spectra of samples dried film (2.0 mg/mL) or in solution (2.0 mg/mL) were collected on a Jasco FT/IR 4100 spectrometer (Easton, MD) in an attenuated total reflection (ATR) mode and using a Ge single-crystal at a resolution of 4 cm^−1^. All the spectral data were processed using built-in software. Spectra were collected in transmission mode and then converted in emission. Each sample was recorded with a total of 100 scans with a rate of 2 mm·s^−1^ against a KBr background.

### Wide-Angle and Small-Angle X-ray Scattering

Fiber diffraction WAXS and SAXS patterns were recorded from dried fibers; WAXS data were measured from water diluted peptide solutions (10–25 mg/mL). Stalks were prepared by the stretch frame method^39^. Briefly, a droplet (10 μL) of peptide aqueous solution (3 wt%) was suspended between the ends of a wax-coated capillary (spaced 2 mm apart). The droplet was allowed to dry gently at room temperature overnight to obtain oriented fibers. Peptide solutions were loaded into Lindemann capillaries and measured, in the same experimental conditions adopted for the dried fibers, at room temperature. WAXS/SAXS data were collected at the X-ray MicroImaging Laboratory (XMI-L@b) equipped with a Fr-E+ SuperBright rotating anode copper anode microsource (Cu K_α_, λ = 0.15405 nm, 2475 W), a multilayer focusing optics (Confocal Max-Flux; CMF 15–105) and a SAXS/WAXS three-pinhole camera (Rigaku SMAX-3000)^40^. For WAXS data collection an image plate (IP) detector with 100 μm pixel size was placed at 8.5 cm from the sample and calibrated by means of the Si NIST standard reference material (SRM 640 b); for SAXS data collection a Triton 20 gas-filled photon counter detector with 200 μm pixel size was placed at 2.2 m from the sample and calibrated by means of silver behenate. A detailed description of the XMI-L@b performances can be found in Altamura *et al*.^40^ and Sibillano *et al*.^41^.

## Additional Information

**How to cite this article**: Diaferia, C. *et al*. Self-assembly of PEGylated tetra-phenylalanine derivatives: structural insights from solution and solid state studies. *Sci. Rep*. **6**, 26638; doi: 10.1038/srep26638 (2016).

## Supplementary Material

Supplementary Information
