# Supplementary material for: Self-assembly of PEGylated tetra-phenylalanine derivatives: structural insights from solution and solid state studies
Source: Sci Rep. 2016 May 25;6:26638. doi: 10.1038/srep26638 (PMC4879547; doi:10.1038/srep26638)

Supporting Information

Self-assembly of PEGylated tetra-phenylalanine derivatives: structural insights from solution and solid state studies

Carlo Diaferia,[1,2] Flavia Anna Mercurio,[2] Cinzia Giannini,[3] Teresa Sibillano,[3] Giancarlo Morelli,[1,2] Marilisa Leone,[2] Antonella Accardo[1,2]

[1]Department of Pharmacy, University of Naples "Federico II" and Interuniversity Research Centre on Bioactive Peptides (CIRPeB), via Mezzocannone 16, 80134 Napoli (Italy)
[2]Institute of Biostructure and Bioimaging (IBB), CNR, via Mezzocannone 16, 80134 Napoli (Italy)
[3]Institute of Crystallography (IC), CNR, Via Amendola 122, 70126 Bari (Italy)

Supporting Table S1. ^{1}H chemical shifts (ppm) for DOTA-L_6-F4 (0.45 mg/mL-340 µM) at 298 K and 600 MHz.

	HN	Hα	Hβ	Aromatic H
F1	8.05	4.50	2.91-2.83	Hδ 7.14<br>Hε 7.30
F2	7.99	4.47	2.88-2.80	Hδ 7.09<br>Hε 7.27
F3	7.95	4.46	2.92-2.88	Hδ 7.15<br>Hε 7.31
F4	7.99	4.46	2.90-3.09	Hδ 7.26<br>Hε 7.34
L6	NHCO**CH2** 2.42<br>NHCOCH2**CH2** 3.61<br>NH**CH2CH2** 3.58, 3.34<br>**NH**CH2CH2 8.01<br>O**CH2CH**2O 3.52, 3.55			
DOTA	3.78, 3.45, 3.38, 3.09, 3.03			

Supporting Figure S1. Secondary structure by chemical shift analysis. Chemical shifts deviations of Hα protons from random coil values $\Delta\delta_{H\alpha} = \delta_{H\alpha observed} - \delta_{H\alpha random\text{-}coil}$

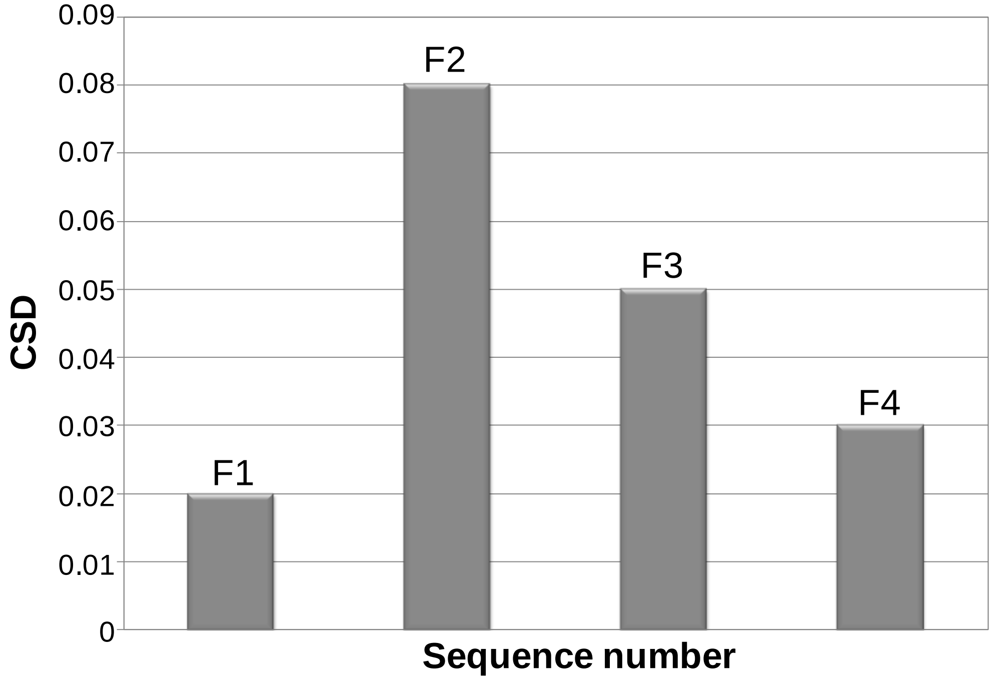

Supporting Figure S2. Comparison of 2D [^{1}H, ^{1}H] TOCSY spectra of L_6-F4 (0.15 mM concentration) (A) and DOTA-L_6-F4 (0.1 mM concentration) (B). The H_N-high field correlation region is shown; in panel (B) an additional amide H_N spin system (indicated by the red arrow), belonging to the linker region L6, that connects the peptide portion to the DOTA ring, can be seen.

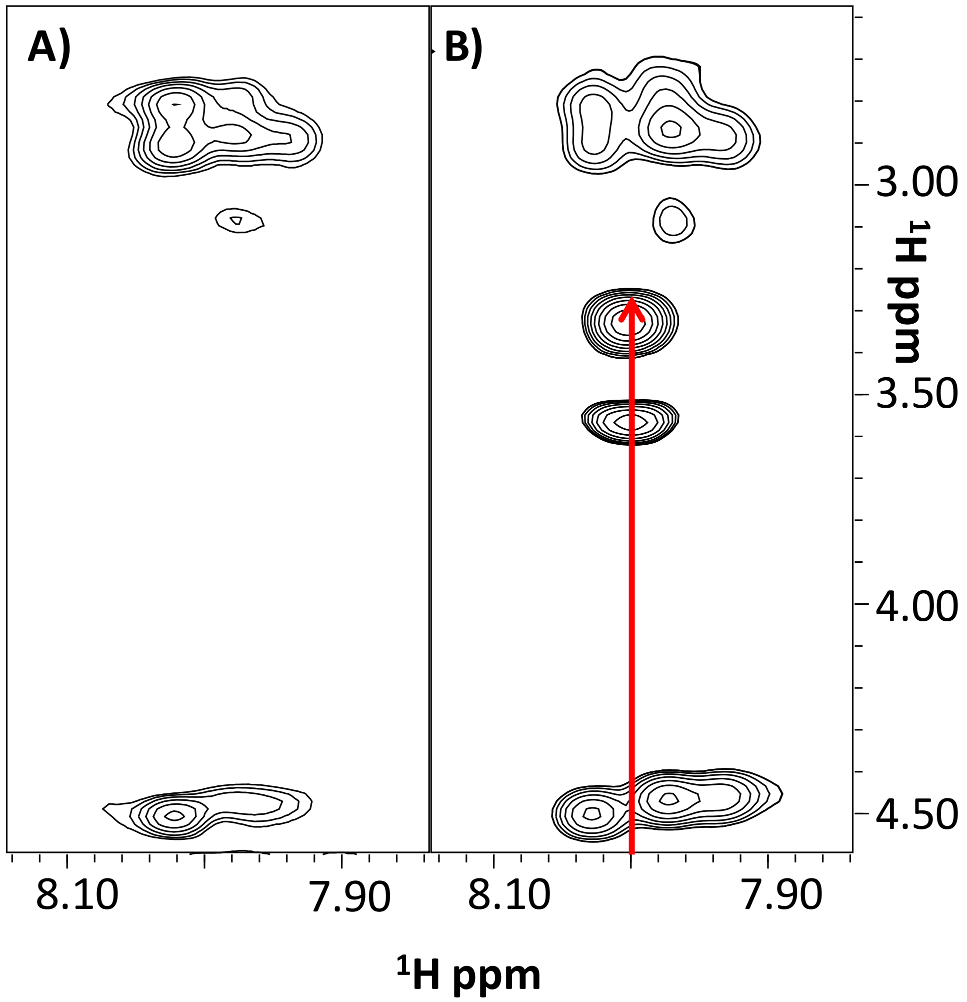

Supporting Figure S3. Intensity correlation functions for: A) DOTA-L_6-F4 (□) and L_6-F4 (■) in aqueous solutions at 1.5 mM concentration; B) L_6-F4 in aqueous solutions at 2.0, 5.0 and 10 mg/mL that correspond to 2.23, 5.58 and 11.2 mM, respectively

A)

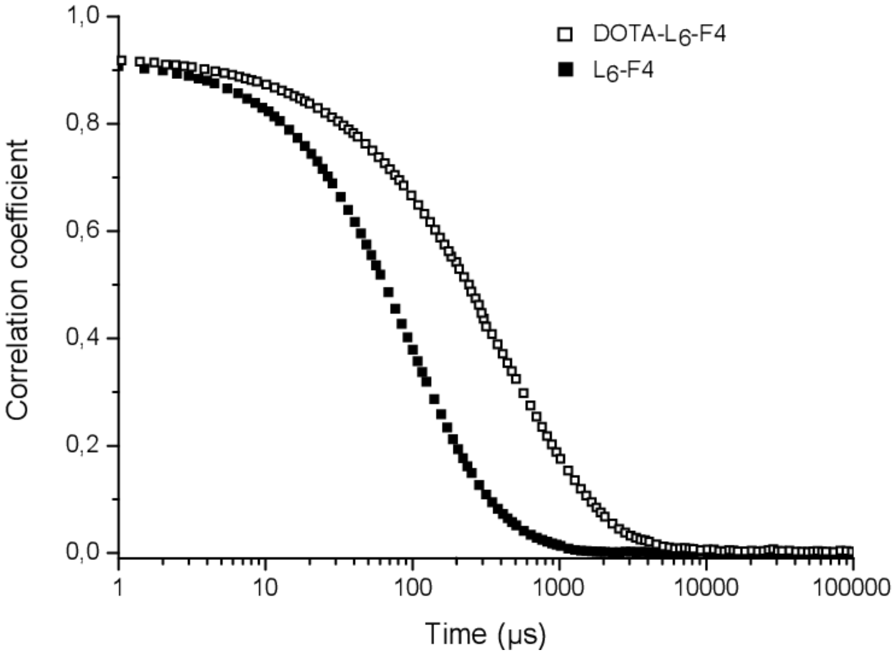

B)

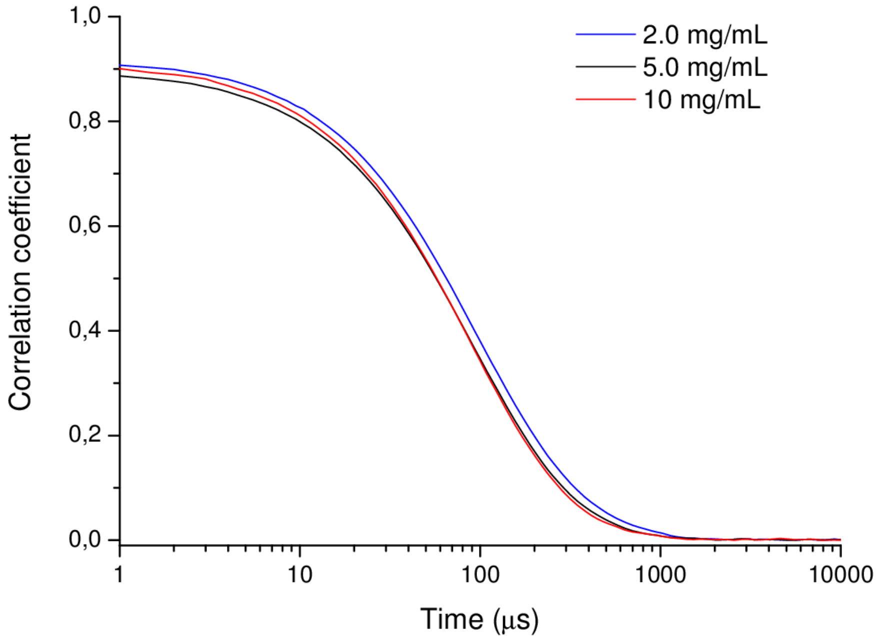

Supporting Figure S4. Simulations of 2D fiber diffraction patterns, computed for increasing fiber disorder: 0.1 rad (5.7°) in (A), 0.2 rad (11.4°) in (B), 0.5 rad (28.6°) in (C) and 0.75 rad (43°) in (D).

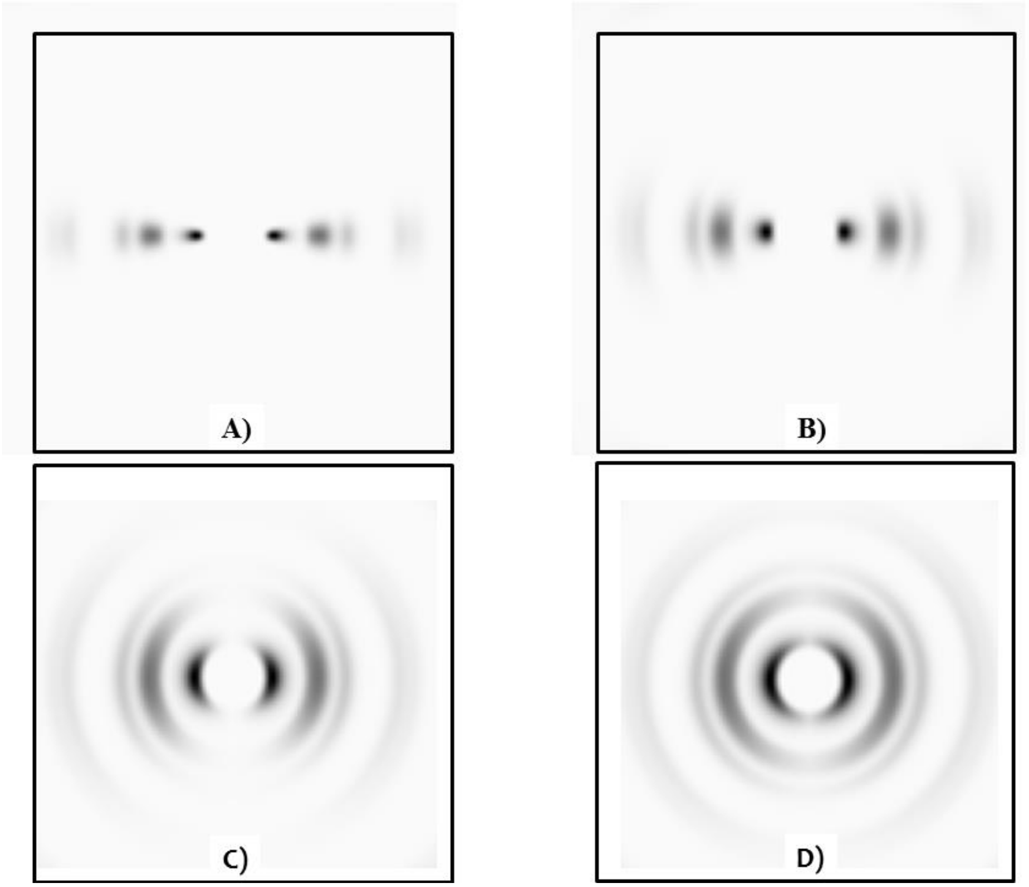

Supporting Figure S5. 2D SAXS data of L_6-F4 (A) and DOTA-L_6-F4 (B) dried fibers. 1D patterns of L_6-F4 (C) and DOTA-L_6-F4 (D).

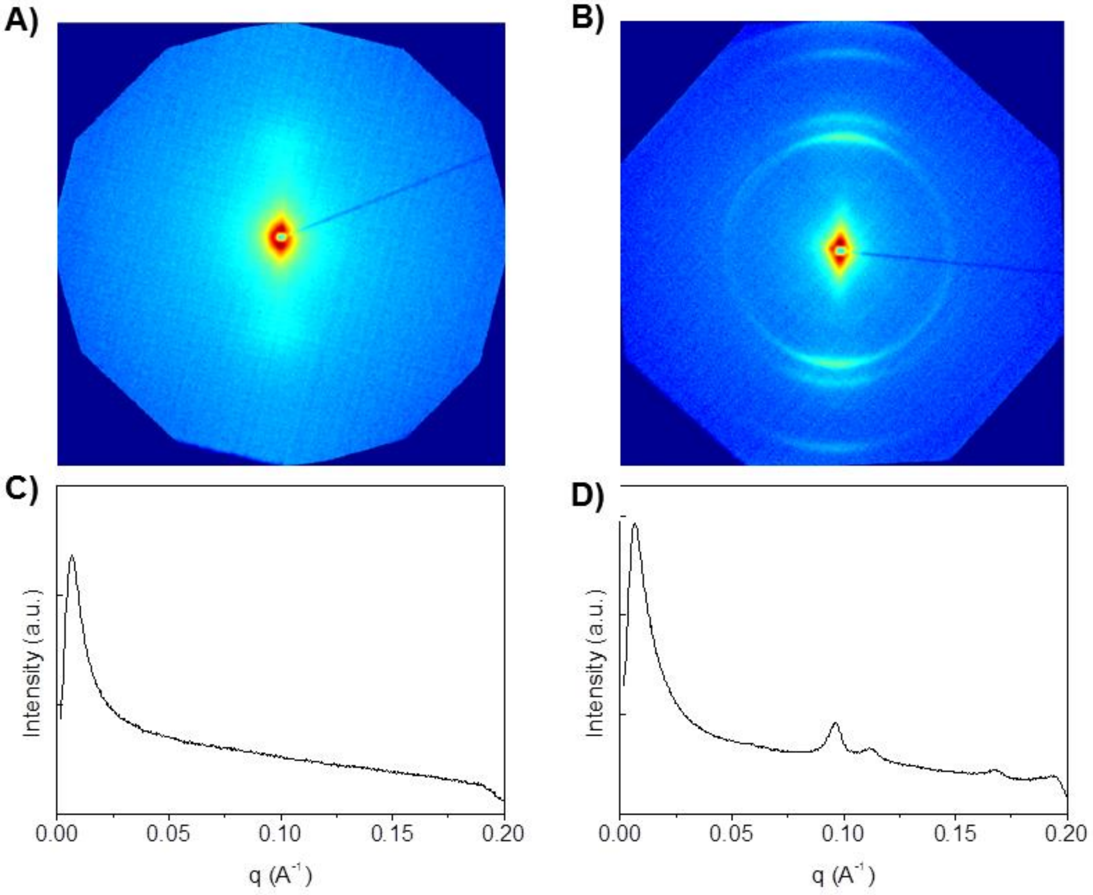

Supplement: Supplementary Information [file srep26638-s1.pdf]
